# Surfing the intelligence with chatbots

**DOI:** 10.4102/sajr.v27i1.2793

**Published:** 2023-11-17

**Authors:** Maya Patel

**Affiliations:** 1 Department of Radiology, School of Medicine, Faculty of Health Sciences, University of the Witwatersrand, Johannesburg, South Africa; 2 National Radiology Services Inc., Johannesburg, South Africa

Instruction-based language model artificial intelligence (AI) programmes like ChatGPT have recently secured a seat at the round table, integrating their role in writing and conversation. The effortless ease with which plausible material can be regurgitated after a few directed commands has been welcomed by users worldwide and created a frenzy in the publishing world.

Many AI tools have become companions in our lives and serve the purpose of simplifying daily tasks. Phones, navigation systems, spellcheckers and word processing programmes, among others, are elements that we can scarcely manage without in the present day. Similarly, language and conversational AI are also likely to become permanently assimilated in our lives and revolutionise the future. However, we need to remain astute of its place in academic literature.

Having blind faith in electronic hardware and the software programs we engineer comes at a price. Concerning issues with large language model AI relate to information accuracy and the analytical validity of the sources used, particularly with scientific writing. There is no control over bias or the references drawn upon. Furthermore, data entered may be retained by the software, threatening confidentiality.

Another alarm is the dilution of the pool of journalistic data as the same information is being exploited to create ‘new’ material. A lack of contributed original work will create a trend towards a negative flux in learning and progress.

From an ethical perspective, probably the most derailing issue is that AI programmes are not authors. In academia, authors are people who receive credit for transparent contribution to a paper and are liable for the work. While AI may be a contributor, it cannot be held accountable for scientific work.

Weighing up the advantages and disadvantages of this situation, it is clear that totally excluding AI is the less favoured choice. The way forward is to evolve through this metamorphosis with caution. The journal publishers and editors have drafted an updated AI policy [https://aosis.co.za/legal-centre/publication-policies/#1699617493200-fadce5d6-e96b] in line with international standards and are re-reviewing plagiarism reports. Ultimately, the editors and reviewers remain the crucial gatekeepers for scholarly publication integrity.

‘*Artificial intelligence is not a substitute for human intelligence: it is a tool to amplify human creativity and ingenuity*’ – Fei-Fei Li

## Recommended additional resources

International Committee of Medical Journal Editors (ICMJE). Recommendations for the conduct, reporting, editing and publication of scholarly work in medical journals. [updated 2023 May; cited 2023 Oct 14] Available from: https://www.icmje.org/recommendations/

Thorp H. ChatGPT is fun, but not an author. Science. 2023;379(6630):313. https://doi.org/10.1126/science.adg7879

SciELO South Africa - SciELO 25 Years Seminar. Technological innovations in scholarly communication. [updated 2023 June 29; cited 2023 Nov 06]. Available from: https://25.scielo.org/en/seminars/south-africa-ai/

COPE. Artificial intelligence and peer review. [cited 2023 Nov 06]. Available from: https://publicationethics.org/publication-integrity-week-2023/ai-peer-review

